# Comparison of formula and number-right scoring in undergraduate medical training: a Rasch model analysis

**DOI:** 10.1186/s12909-017-1051-8

**Published:** 2017-11-09

**Authors:** Dario Cecilio-Fernandes, Harro Medema, Carlos Fernando Collares, Lambert Schuwirth, Janke Cohen-Schotanus, René A. Tio

**Affiliations:** 1 Center for Education Development and Research in Health Professions (CEDAR), University of Groningen and University Medical Center Groningen, Antonius Deusinglaan 1, FC40, 9713 AV Groningen, The Netherlands; 2 Department Business IT & Management, NHL University of Applied Sciences, Leeuwarden, Netherlands; 3 Faculty of Health, Medicine and Life Sciences, Educational Development and Research, Maastricht University, Maastricht, Netherlands; 4 Prideaux Centre for Research into Health Professions Education, Flinders University, Adelaide, Australia

**Keywords:** Assessment, Multiple choice questions, Formula scoring, Number-right scoring, Rasch model, Reliability, Validity, Construct-irrelevant variance

## Abstract

**Background:**

Progress testing is an assessment tool used to periodically assess all students at the end-of-curriculum level. Because students cannot know everything, it is important that they recognize their lack of knowledge. For that reason, the formula-scoring method has usually been used. However, where partial knowledge needs to be taken into account, the number-right scoring method is used. Research comparing both methods has yielded conflicting results. As far as we know, in all these studies, Classical Test Theory or Generalizability Theory was used to analyze the data. In contrast to these studies, we will explore the use of the Rasch model to compare both methods.

**Methods:**

A 2 × 2 crossover design was used in a study where 298 students from four medical schools participated. A sample of 200 previously used questions from the progress tests was selected. The data were analyzed using the Rasch model, which provides fit parameters, reliability coefficients, and response option analysis.

**Results:**

The fit parameters were in the optimal interval ranging from 0.50 to 1.50, and the means were around 1.00. The person and item reliability coefficients were higher in the number-right condition than in the formula-scoring condition. The response option analysis showed that the majority of dysfunctional items emerged in the formula-scoring condition.

**Conclusions:**

The findings of this study support the use of number-right scoring over formula scoring. Rasch model analyses showed that tests with number-right scoring have better psychometric properties than formula scoring. However, choosing the appropriate scoring method should depend not only on psychometric properties but also on self-directed test-taking strategies and metacognitive skills.

## Background

Progress testing is a systematic, longitudinal assessment method, by which students are periodically assessed at end-of-curriculum level. Research has shown that the progress test is a valid and reliable tool for measuring knowledge growth [1–3], it reduces examination stress, and it positively influences student learning [4].

Over the past few decades, test scores on assessment tools based on multiple-choice questions (MCQs) have been calculated in two ways: “number-right scoring” and “formula scoring.” Number-right scoring implies that only the number of correct answers is taken into account when calculating the total score, and that incorrect answers are not subtracted from the total score. Number-right scoring has frequently been applied for a number of reasons. First, its simplicity allows for an uncomplicated interpretation of the results for both students and professionals. Second, number-right scoring allows students to answer all questions, and their partial knowledge is included in the outcomes. If students have partial knowledge about an item and can rule out alternatives with more or less certainty, they will obtain higher scores [5]. Third, under the presumption that the test tries to measure the knowledge a student has and not just the knowledge that they are confident in using, the willingness to guess is not accounted for in number-right scoring, which reduces bias regarding construct-irrelevant sources of variance due to risk-avoidance behavior.

Although formula-scoring method tests are not frequently used, except for progress tests in medicine, it gives students the opportunity to acknowledge that they do not know the correct answer instead of forcing them to guess [6]. It is important to realize that students cannot know everything. Due to the different knowledge levels of the participating students in the case of progress testing, the inclusion of an “I don’t know” option becomes a logical choice. In progress tests using formula scoring, an “I don’t know” option – which does not lead to a penalty – is included. When such a scoring method is applied, junior students tend to answer a smaller percentage of the questions than senior ones. Formula scoring offers an individualized way of correction for guessing and may reduce random guessing to as low as 2% of the items [7].

Comparisons between number-right scoring and formula scoring have been the subject of study for many years. Data comparing the reliability of both methods have yielded conflicting results. Formula scoring has shown an increase [6, 8] and a decrease [9] in the reliability coefficient as compared to number-right scoring. This increase in reliability, however, might be related to other constructs that are reflected in the final score [10–12], such as risk-taking strategies [6, 13–15], gender [16–19], self-efficacy beliefs, and metacognitive skills, instead of students’ medical knowledge alone [6, 20, 21]. From a practical perspective, one could argue that knowledge is only useful if the student is willing to use it and that focusing only on the knowledge in the ‘heads’ of students might be a case of construct-underrepresentation. Furthermore, students have differed in their tendency to choose the “I don’t know” option [17, 19, 22].

This study aims to answer the following research questions:

- Which scoring method provides fewer dysfunctional items?
- Which scoring method provides the most reliable score?

Traditionally, Classical Test Theory (CTT) and Generalizability Theory analyses have been used to investigate differences between number-right and formula scoring [6, 8, 9]. In contrast to these previous studies, we have based our data analyses on Item Response Theory (IRT). IRT was chosen because it allows for an estimate of student ability (theta) that is independent of item selection; moreover, item difficulty (b) can be estimated in a way that is independent of the sample of students. These two properties are called parameter invariance. Additionally, IRT provides an estimate of the measurement error at each point of the *theta* (ability), which allows for an estimation of the reliability of each student’s performance. Despite evidence of the advantages of IRT models over CTT [23], it is only possible to take full advantage of IRT if two assumptions are met. The first assumption is unidimensionality, which implies that a single underlying trait accounts for the performance of the student. The second assumption is local independence, which implies that test items cannot be related to each other [24]. For more information about IRT and the comparison between IRT and CTT, see Downing (2003) [25] and De Champlain (2010) [26]. Since IRT models are more sensitive to construct-irrelevant sources of variance, we expected that the tests taken using the number-right scoring condition would be more reliable and have better validity. In addition, fewer dysfunctional items should emerge for the tests that use the number-right scoring condition.

## Methods

To answer our research questions, an experiment was designed comparing the number-right and formula-scoring methods using a 2 × 2 crossover design (Table 1). For the first test of group A, formula scoring was used and, for the second, number-right scoring, whereas group B was tested the other way around. This design avoided cueing and priming effects, and ensured similar student knowledge levels.

**Table 1:** Crossover design of tests 1 & 2 versus groups A & B with formula-scoring and number-right scoring conditions per year

	Group A *n* = 153	Group B *n* = 145
Test 1	Formula scoring (FS)	Number-right scoring (NR)
Test 2	Number-right scoring (NR)	Formula scoring (FS)

### Participants and procedure

Medical students from years 2, 3, and 4 were invited to participate in the experiment. Unlike year-one students, their knowledge levels were expected to be sufficient to provide useful information, and they would then be more likely to make an educated guess instead of not answering an item (the “I don’t know” option). Additionally, years 2, 3, and 4 medical students were chosen because they were in a structured learning environment, where there was likely to be more homogeneity in the cohorts in terms of educational experience. Two hundred ninety-eight students from four Dutch medical schools participated in the experiment (Table 1).

In this particular research field, it is important for the participating students to already be acquainted with the blueprint and the test format. Our participants were familiar with both types of questions and scoring methods, since they had taken both kinds of tests at least five times. This provided a methodological advantage that enabled us to better establish construct validity through the comparison of scores, minimizing measurements of other traits [5, 11].

### Instruments

The Dutch progress test covers the whole domain of medical knowledge at end level, based on the Dutch National Blueprint for the Medical Curriculum. The progress test is simultaneously administrated four times a year to all medical students who take part in the consortium. At that time, roughly 10,000 students take the progress test. Each progress test consists of 200 multiple-choice questions. Since 2005, the Dutch Interuniversity Progress Test has comprised items with a varying number of response options, ranging from 2 to 5. The penalty for guessing for each item varies according to the number of distracters (−1/[the number of answer options-1]), ranging from −1.00 to −0.25.

We selected 250 questions out of seven progress tests that had been administered between 2005 and 2007. Subsequently, we reduced the number of questions to 200 items with a *p*-value > .25, indicating the probability of the question being answered correctly in a cohort of students. We created two equal tests of 100 multiple-choice questions, based on the progress test blueprint. Both sets of 100 questions were equally distributed in terms of mean *p*-values, based on the results of graduate level students, through use of the sum of *p*-values, the sum of p-corrected, the total of “I don’t know” options chosen, and the total number of distractors per question (2, 3, or 4). All those statistics are based on Classical Test Theory and were gathered from the quality control of the Dutch progress test consortium.

Students were divided into two groups: Group A took the first set of 100 items under formula-scoring conditions and group B the same items under number-right scoring conditions. For the second set of 100 items, it was the other way round: group A under number-right scoring conditions and group B under formula-scoring conditions. For the test using formula-scoring, students could choose an “I don’t know” option. For the test using the number-right scoring, the “I don’t know” option was not available, and students had to give an answer. An example of a question in the formula-scoring test is:

In patients with hydrocephalus, the cerebrospinal fluid is in most cases re-routed through a shunt system from the lateral ventricles

- To the venous system
- To the thoracic duct
- To the peritoneal cavity
- To the spinal cord
- I don’t know

The same question was in the number-right test.

In patients with hydrocephalus, the cerebrospinal fluid is in most cases re-routed through a shunt system from the lateral ventricles

- To the venous system
- To the thoracic duct
- To the peritoneal cavity
- To the spinal cord

### Data analysis based on item response theory (IRT)

There are several IRT models available, but the Rasch model was used for several reasons. First, it is a simpler and stricter model than the 2-parameter and the 3-parameter logistic models, which means that the Rasch model is more susceptible to a violation of the data than the 2-parameter and the 3-parameter logistic models [26, 27], thus allowing dysfunctional items to be identified. The Rasch model requires a smaller sample size. For a two-tailed 99% confidence interval, the minimum sample size is 108 subjects [28]. Furthermore, it is widely used in medical education [29–33].

### Preliminary analysis

Unidimensionality was tested using the Principal-Components Analysis of Residuals (PCAR) and a fit-only approach [34]. The latter has two fit parameters for person and item. Whereas *infit* excludes the outliers from the analysis, *outfit* includes the outliers from the analysis. Both *infit* and *outfit* were calculated using the mean square (MS). The optimal fit value is 1.00 [35] with a range from .50 to 1.50 [36] for both the person and the item. However, violations of the fit parameter for a person are better tolerated and expected, whereas items with *infit* and *outfit* higher than 2.0 are a threat to the validity of the test [36] and are recommended for exclusion.

For the Principal-Components Analysis of Residuals, we first considered whether another dimension would have more than two items. If so, we further investigated the amount of explained variance. Correlation of the standardized residual was calculated to check the local independency. If items present a correlation lower than 0.7, the local independency assumption holds.

### Linking and equating

Linking and Equating was not deemed necessary, because both groups answered the same multiple-choice questions. Our 2 × 2 crossover design (Table 1) ensured similar student knowledge levels in both scoring methods, which controlled for guessing and discrimination of the items throughout the groups. Furthermore, a post analysis of the level of students’ ability revealed no significant difference between students in Tests 1 and 2 (*t* = 1.803, *p* = 0.07 and *t* = 1.771, *p* = 0.08, respectively). Since the data were analyzed using the Rasch model, which has the property of parameter invariance, all four groups were comparable.

### Calibration of the Rasch models

The four tests were analyzed and calibrated separately, since we were interested in comparing the psychometric properties of both scoring methods. Because of that, the most appropriate Rasch model for each condition needed to be chosen. For formula scoring, we used the Rasch Partial Credit model for polytomous categories, since the categories follow an ordinal arrangement with the right answer having the highest (5), the question mark having the second highest (4), and the penalties having the lowest values, representing the amount of penalty (3, 2, and 1). The penalty was recoded according to the number of distractors. Items with two-options answers were recorded as one; three-option items were recoded as two; and four options as three, since the penalty is higher in cases of fewer distractors. For the number-right scoring, we used the Rasch dichotomous model. All data were analyzed using Winsteps 3.70.1.1 (Winsteps Rasch Measurement 2009).

To answer our first research question, the response-option analysis was conducted to evaluate the average ability for each response option. This analyzes the appropriate category order (whether the category of polytomous items is ordered as expected).

To answer our second research question, we calculated two reliability coefficients based on the Rasch, one for the person and another for the item. The latter is an indication of sample size. Low item reliability means that the sample size is not large enough to estimate the parameters. The person reliability is equivalent to the traditional test reliability (e.g., Kuder-Richardson-20, Cronbach’s alpha); low values can indicate a small number of items or a narrow range of person measurements. The person reliability coefficient is calculated using measurement standard errors.

## Results

First, we will describe the analyses of dimensionality, fit parameter, and local independence. After that, we will present the Rasch reliability coefficients for person and item. Finally, we will describe the dysfunctional items.

### Preliminary analysis

The four tests had three or four items in the first contrast, which could indicate a second dimension. The variance explained by the items in the number-right scoring condition was higher than five times the variance explained by the first contrast: 17.9% vs. 3.3%. In addition, the explained variance in the first contrast was smaller than the variance explained by persons and items. Comparable values were found for the items in the formula-scoring condition: The explained variances were 17.9 and 3.7% for the first contrast.

Regarding the items, the fit parameters were in the optimal interval from 0.50 to 1.50 [36], and the means were near 1.00, which is the optimal value for the *infit* and *outfit*. Mean, standard deviation, minimum and maximum of measurement, *infit*, *outfit*, and error based on Rasch outcomes are shown in Table 2.

**Table 2:** Mean, standard deviation, minimum and maximum of measurement, infit, outfit, and error for items and person

			Items				Person			
			Measure	*Infit*	*Outfit*	Error	Measure	*Infit*	*Outfit*	Error
Test 1	FS	Mean	0.00	1.00	1.00	0.12	0.32	1.04	1.00	0.13
		SD	0.55	0.05	0.10	0.05	0.25	0.37	0.41	0.01
		Minimum	−1.85	0.91	0.52	0.05	−0.38	0.41	0.36	0.11
		Maximum	1.55	1.22	1.48	0.38	1.54	2.51	2.21	0.21
	NR	Mean	0.00	1.00	1.00	0.20	0.24	1.00	1.00	0.23
		SD	1.17	0.06	0.10	0.07	0.50	0.09	0.20	0.01
		Minimum	−4.15	0.84	0.79	0.17	−1.32	0.81	0.72	0.22
		Maximum	2.57	1.12	1.37	0.71	1.57	1.29	2.58	0.26
	FS	Mean	0.00	1.00	1.03	0.12	0.33	1.03	1.03	0.12
Test 2		SD	0.59	0.05	0.14	0.05	0.25	0.35	0.44	0.01
		Minimum	−2.68	0.90	0.83	0.05	−0.31	0.27	0.34	0.11
		Maximum	1.71	1.25	2.02	0.39	1.27	2.14	3.46	0.18
	NR	Mean	0.00	1.00	0.99	0.18	0.24	1.00	0.99	0.22
		SD	0.90	0.06	0.10	0.03	0.51	0.10	0.15	0.01
		Minimum	−2.32	0.85	0.66	0.17	−1.53	0.78	0.66	0.22
		Maximum	2.42	1.13	1.19	0.30	1.75	1.32	1.53	0.27

*FS* formula-scoring group, *NR* number-right scoring group

There was only one item in the formula-scoring condition of group B that had *outfit* higher than 2.00. Regarding the person parameters, there were some violations of the maximum and minimum value of the recommended interval, especially in the formula-scoring condition.

Regarding local independency, the highest correlation of the standardized residual was 0.35. If items present a correlation lower than 0.7, the local independency assumption holds. Locally dependent items are considered as threats to unidimensionality [24, 25].

### Which scoring method provides fewer dysfunctional items?

There was a clear difference in numbers of dysfunctional items between the formula-scoring and number-right tests. Most dysfunctional items were found (1) when participants in the question-mark category had higher or equal ability versus those in the right-answer category (*n* = 7) and (2) when participants in the penalty category had higher ability versus those with a correct answer or a question mark (*n* = 25). For both groups in the number-right condition, (1) 5 items had the higher ability in the wrong category, and (2) one item had the same ability between the right and wrong categories. Table 3 summarizes the dysfunctional items in terms of the relationship between ability and category.

**Table 3:** Differences between formula score and number right from a Rasch perspective, influence on items

		W > R	W = R	? = P	? > R	? = R	P >?	P >?;R	Total
Test 1	FS	NA	NA	3	5	0	6	2	16
	NR	5	1	NA	NA	NA	NA	NA	6
Test 2	FS	NA	NA	1	1	1	11	6	20
	NR	5	1	NA	NA	NA	NA	NA	6

*FS* formula-scoring group, *NR* number-right scoring group, *W* Wrong, *R* Right,? Question Mark, *P* Penalty. Count, *NA* not applicable

Based on these findings, all dysfunctional items were excluded from the model in terms of further analysis. After the exclusion of items, the variance explained by the items increased, and the fit parameters were in the optimal interval. There was no item with an *infit* or *outfit* above 2.0.

### Which scoring method provides the most reliable score?

Interestingly, the reliability coefficients for person were higher after the exclusion of the items, whereas the reliability coefficients for the items were similar for both scoring methods. After the exclusion, the Rasch reliability coefficients for person and item for each test are shown in Table 4. The reliability coefficients ranged from 0.73 to 0.82 for the persons and from 0.94 to 0.96 for the items. The item reliability coefficients were comparable in both conditions. However, the person reliability coefficients were higher in the number-right (0.80 and 0.82) than in the formula-scoring condition (0.73 and 0.77) on Tests 1 and 2, respectively.

**Table 4:** Person and Item reliability coefficient per test based on the Rasch

	Test 1		Test 2	
	FS	NR	FS	NR
Person reliability	0.73	0.80	0.77	0.82
Item reliability	0.94	0.96	0.94	0.96

*FS* formula-scoring group, *NRB* number-right scoring group

**Fig. 1:**
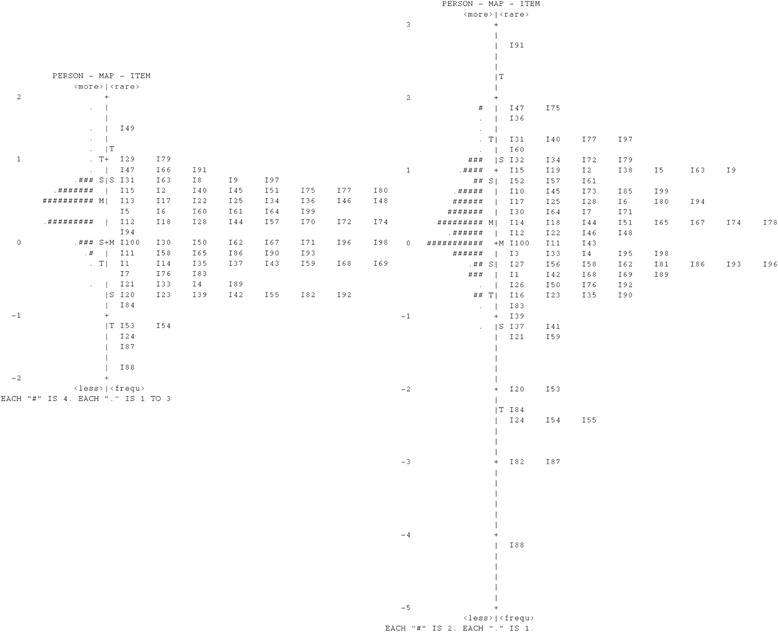
Map of question difficulty and student ability for Test 1. Left hand side shows questions under the formula scoring method and the right hand side shows questions under the right number scoring method

**Fig. 2:**
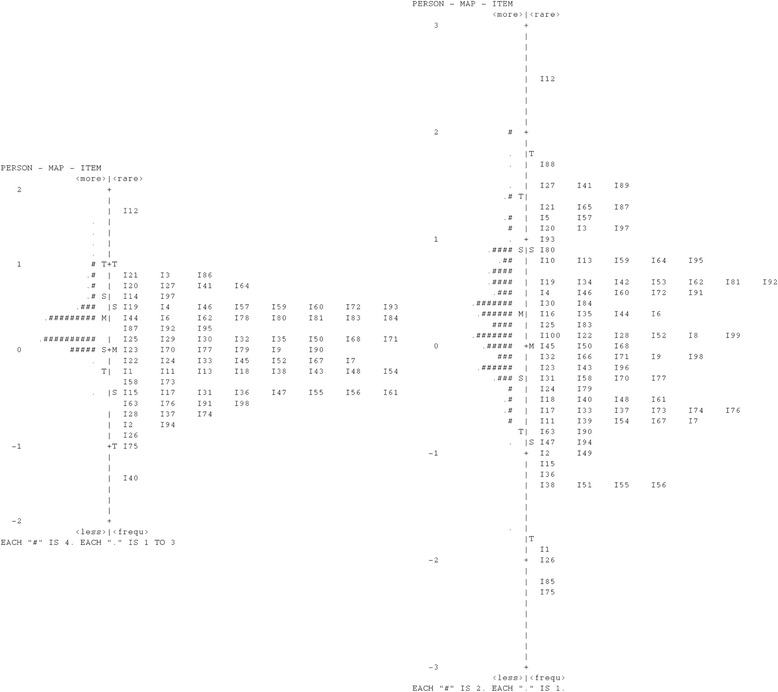
Map of question difficulty and student ability for Test 2. Left hand side shows questions under the formula scoring method and the right hand side shows questions under the right number scoring method

In Figs. 1 and 2, the influence of both the scoring methods on the same items is visualized in Tests 1 and 2. As is visualized at the left side, the items using the formula-scoring method ranged from −2 to 2 logit for both tests, while the items using the number-right scoring method ranged from −5 to 3 and −3 to 3 logit. The items using formula scoring varied less in terms of difficulty than the items using number-right scoring, resulting in lower discrimination regarding student ability. Because of that, the students subjected to number-right scoring could be better differentiated in both tests than those students subjected to formula scoring. The difference in variability also explains why the reliability for number-right scoring was higher than for formula scoring.

## Discussion

In this study, the Rasch model methodology was used to investigate whether number-right or formula scoring should be preferred for progress testing. The outcomes of the Rasch model analysis showed that item-reliability coefficients were comparable. Number-right scoring presented higher person reliability coefficients and fewer dysfunctional items than formula scoring.

Our methodology and findings differ from previous studies in several ways. The 2 × 2 crossover design is especially useful for avoiding cueing and priming effects during data collection. Moreover, we ensured that all students answered different tests in both conditions, which allowed us to assume similar knowledge levels in both conditions. Another methodological difference was the use of the Rasch model. To our knowledge, this has not been done in previous studies. Regarding our results, two main findings emerged. First, person reliability coefficients, which are similar to CTT reliability coefficients, were clearly higher for number-right scoring for both tests, which contradicts some previous studies [6, 8]. Higher person reliability indicates that the test can differentiate better between levels of student ability and that obtaining the same ordering of students using repeated measurements is more likely [35]. This study shows that it is possible to obtain higher reliability coefficients with fewer items when the Rasch model is used. Further studies are necessary to investigate whether our findings are transferable to other years in medical school.

Second, the response options analysis showed clear differences between number-right scoring and formula scoring. The formula-scoring tests produced around three times more dysfunctional items. In theory, the question-mark category could have higher ability averages, since students who know the content would also be aware of what they do not know. However, the highest number of dysfunctional items emerged when students in the penalty category had a higher average ability than students in the right or question-mark categories. At the same time, our results showed that there were only two items that were dysfunctional in both scoring conditions. Therefore, we believe that formula scoring could be a possible source of dysfunctionality. To our knowledge, this is the first study to indicate that formula scoring may possibly be a contributing factor in this phenomenon. Further studies are necessary to investigate whether formula scoring contributes to item misfit.

Some limitations have to be considered. Students’ test-taking strategies may change after a series of tests. In this particular study, however, students were already acquainted with both scoring methods. The second limitation may be that the experimental setting is somewhat artificial. In reality, the progress test is a mix of summative and formative formats, so the scores in our study may be biased by the students’ willingness to participate. The formative format allows students to receive feedback without the risk of being categorized. A summative decision is only made after a serious of progress tests. Third, there may be small recognition effects due to our item sample. Some of the students may have answered some of the questions three or more years earlier. The final limitation may be that the reliability estimates could not be compared between years of medical school separately.

Despite the importance of the psychometrics properties of a test, other aspects should be taken into consideration, especially because the progress test is just one of the many assessment tools that are used to evaluate student learning. Since we do not expect junior students to be able to answer all questions, the inclusion of an “I don’t know” option becomes a logical choice. However, a recent study has demonstrated that students in the later years are more likely to guess and actually answer a question incorrectly than first-year medical students [37], which raises the question of the educational purpose of the “I don’t know” option. At the same time, formula scoring may penalize students with more knowledge, since they are less likely to guess and so do not answer items that they only have partial knowledge about [11]. Additionally, the use of formula-scoring causes bias due to both item-specific and systematic willingness to guess. Item-specific means that students weigh the penalty for an incorrect answer against the probability of a correct answer [38]. Systematic willingness to guess means that some students are more willing to guess than others, for example, male students appear to guess more often than female students [16]. Formula scoring may encourage students to use self-directed test-taking strategies. This may happen, for example, if an item has a higher penalty, because it has fewer response options. Whether a student will answer an item will therefore not just depend on the student’s estimate of the probability of answering the item correctly but also on the risk-avoidance behavior of the student [14]. This may introduce noise into the test, since the score variance may also be influenced by self-efficacy beliefs and metacognitive skills instead of students’ medical knowledge alone [6, 20, 21]. Our finding that the person reliability coefficient is lower in the formula-scoring condition supports these considerations. It is, however, encouraging that the item reliability coefficients of both conditions were similar in terms of the impact of formula scoring on students’ learning behavior. Future studies are necessary in order to investigate whether the use of the “I don’t know” option leads to increased self-efficacy beliefs. Further research on the use of Rasch analysis for progress testing is still necessary, especially taking into account the longitudinal character of the test.

## Conclusions

Rasch model analyses showed that number-right tests have better psychometric properties than formula scoring. Based on our psychometric analysis alone, the use of the number-right scoring method seems logical for multiple-choice question tests.

## Abbreviations

?: Question mark
CTT: Classical test theory
FSA: Formula-scoring group A
FSB: Formula-scoring group B
IRT: Item response theory
NA: Not applicable
NRA: Number-right scoring group A
NRB: Number-right scoring group B
P: Penalty
R: Right
SD: Standard deviation
W: Wrong
